# Microwave-assisted extraction of phytochemicals from *Piper betle* L.: Optimization, characterization, and bioactivity evaluation^☆^

**DOI:** 10.1016/j.fochx.2025.102672

**Published:** 2025-06-17

**Authors:** Tripti Singh, Zaryab Shafi, Rahul Singh, Bhawna Bisht, Krishna Kumar Yadav, Jari S. Algethami, Ghadah Shukri Albakri, Maha Awjan Alreshidi

**Affiliations:** a Department of Biosciences, Integral University, Lucknow, Uttar Pradesh 226026, India; b Department of Bioengineering, Integral University, Lucknow, Uttar Pradesh 226026, India; c Department of Food Science & Technology, Graphic Era (Deemed to be University), Dehradun 248002, India; d Department of VLSI Microelectronics, Saveetha School of Engineering, Saveetha Institute of Medical and Technical Sciences (SIMATS), Saveetha University, Chennai-602105, Tamil Nadu, India; e Environmental and Atmospheric Sciences Research Group, Scientific Research Center, Al-Ayen University, Nasiriyah, Thi-Qar 64001, Iraq; f Department of Chemistry, College of Science and Arts, Najran University, P.O. Box 1988, Najran 11001, Saudi Arabia; g Advanced Materials and Nano-Research Centre (AMNRC), Najran University, Najran 11001, Saudi Arabia; h Department of Teaching and Learning, College of Education and Human development, Princess Nourah bint Abdulrahman University, P.O. Box 84428, Riyadh 11671, Saudi Arabia; i Department of Chemistry, College of Science, University of Ha'il, Ha'il 81441, Saudi Arabia

**Keywords:** *Piper betel* L., Microwave-assisted extraction (MAE), Response surface methodology (RSM), Bioactive compounds, Antioxidant activity, antibacterial activity

## Abstract

This study aimed to enhance the phytochemical yield from *Piper betel* L. leaves by optimizing microwave-assisted extraction (MAE) parameters using response surface methodology (RSM). The optimized conditions were found to be 239.6 W (microwave power), 1.58 min (extraction time), and 1:22 (solid-to-solvent ratio). Under these conditions, the extract yield was 8.92 %, with a total phenolic content (TPC) of 77.98 mg GAE/g, total flavonoid content (TFC) of 38.99 mg QUE/g, antioxidant activity of 62.95 %, and chlorophyll content of 42.02 mg/mL. Gas chromatography-mass spectrometry (GC–MS) analysis identified key bioactive compounds, including phytol and neophytadiene. Structural analysis by scanning electron microscopy (SEM) revealed substantial cellular disruption, confirming efficient extraction. Furthermore, the extract demonstrated strong antibacterial activity against *E. coli*, *B. cereus*, *K. pneumoniae*, and *B. pumilus*. These findings underscore the effectiveness of optimized MAE in extracting potent phytochemicals from betel leaves, reinforcing its potential in natural product development and therapeutic applications.

## Highlights

• Optimized MAE enhanced yield (8.92 %) & bioactive content from betel leaves.
• FTIR & GC–MS identified key antioxidants like phytol & neophytadiene.
• Strong antibacterial activity against *E. coli, B. cereus, K. pneumoniae* & *B. pumilus*.
• MAE offers an eco-friendly, cost-effective alternative for bioactive extraction.

## Graphical abstract

**Figure:**
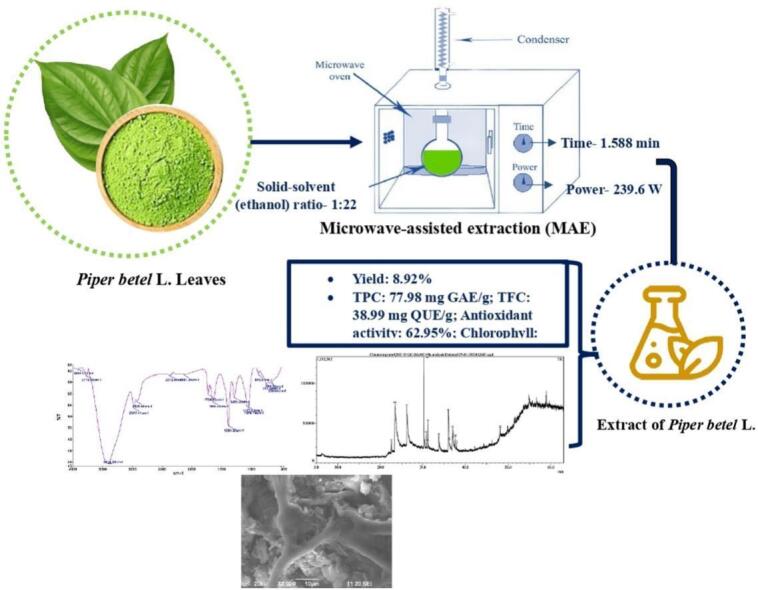

## 1 Introduction

Medicinal food plant extracts, rich in bioactive non-nutrient phytochemicals, have been foundation of folk phytomedicine for conventional healing practices due to their natural origin, low toxicity levels, therapeutic potential (Gupta et al., 2023; Twaij & Hasan, 2022). Among these, *Piper betle* L. (Family: *Piperaceae*), an evergreen perennial vine widely cultivated in Southeast Asia, has garnered significant attention for its medicinal properties (Azahar et al., 2020; Muruganandam et al., 2017). In India, it is popularly known as Paan and ranks just after tea and coffee in terms of daily consumption (Muruganandam et al., 2017). Betel leaf possesses several beneficial properties, including nutritional, organoleptic, medicinal, prophylactic and functional (Madhumita et al., 2020). However, scientific research reveals that its potential in therapeutic applications is attributed to its rich profile of bioactive compounds, particularly phenolics, which exhibit anti-mutagenic, anti-tumor, and antioxidant activities, making it a promising candidate for the development of commercial products (Azahar et al., 2020; Muruganandam et al., 2017). Several studies have reported evaluating bioactive components of *Piper betle* L. and extracting and separating useful compounds using various techniques (Abdullah & Mohamad Hussain, 2015; Muruganandam et al., 2017). Nevertheless, the quality and quantity of plant extract bioactive chemicals are highly dependent on the geographical origin and extraction methods utilized on different parts of the plant (Gupta et al., 2023). However, few research studies have focused on maximizing extract yield for various parameters. Selecting an appropriate extraction method is crucial, as each technique has its advantages and limitations. Soxhlet extraction remains one of the most widely used conventional techniques and serves as a benchmark for evaluating other techniques (López-Avilés et al., 2025). However, it has notable drawbacks, including long processing times, high temperatures, excessive solvent use, low selectivity, reduced yields, and potential degradation of thermo-sensitive compounds (Bisht, Gururani, et al., 2023).

To overcome the shortcomings of the soxhlet extraction technique stated above, advances in non-conventional or non-thermal or greener extraction approaches have resulted MAE as a superior technique to other conventional methods. This technique not only accelerates processing by rapidly heating the solvent with electromagnetic energy, but it also improves access to porous materials, solvent penetration, and extraction rates, allowing bioactive compounds to be released from the cell (Azahar et al., 2020; Bagade & Patil, 2021). Thus, the selection of an appropriate solvent based on microwave-absorbing characteristics, analyte selectivity, and solvent-matrix interaction is critical (Azahar et al., 2020). The scientific literature has acknowledged this technique for its capacity to increase product quality while using less solvent and decreasing thermal degradation of products (Singh et al., 2023). However, due to limited investigations into the application of MAE to examine the yield of bioactive compounds from *Piper betel* L. extract (Astyka et al., 2024), highlighting the need for further research on this technique.

Thus, employing mathematical modelling as a key optimization tool, the current work aims to optimize MAE conditions for *Piper betel* L. leaves using RSM by systematically varying microwave power, extraction time, and solid-to-solvent ratio to maximize the yield and recovery of bioactive compounds. Furthermore, the chemical composition, bioactive properties, and structural changes of the extracts will be evaluated using FTIR, GC–MS, and SEM under optimal conditions.

## 2 Materials and methods

### 2.1 Collection of plant material

Fresh betel leaves were picked from Naka Hindola, Lucknow. Damaged leaves were sorted from the obtained leaves, and the dirt and residue were removed with running water. These betel leaves were chopped into small fragments and left to dry overnight in a hot air oven set at 40 °C. The dried leaves were crushed into a powder and hand-screened to a particle size of 150 μm and stored in airtight pouches.

### 2.2 Chemicals and reagents

The study utilized analytical-grade chemicals and reagents, which were sourced from Hi Media.

### 2.3 Preliminary trials

Preliminary trials were conducted to determine appropriate ranges. As summarized **in S Table 1**, various solvents, particle sizes, and sample sizes, MAE conditions including time, power were evaluated to identify conditions that maximize extract yield of *Piper betel* L. Ethanol (95 % *v*/v), a polar solvent capable of dissolving both polar and non-polar compounds due to its hydroxyl group, produced the highest yield. Consequently, particle size of 150 μm, and a sample size of 10 g were selected for subsequent experiments to ensure consistent and efficient extraction.

### 2.4 Experimental design for MAE

MAE was employed to optimize the extraction of phytochemicals from *Piper betel* L. leaves. The extraction parameters included microwave power (180 W, 240 W, and 300 W), extraction time (1 min, 2 min, and 3 min), and solid-to-solvent ratio (1:10, 1:20, and 1:30). A total of 17 experimental runs were performed based on the Box-Behnken Design (BBD) using RSM. Each experiment was conducted in triplicate, and the conditions were randomized using Design-Expert Software (Singh et al., 2024). Following the extraction process, the solvent was evaporated using rotary evaporator under reduced pressure at 40 °C until a concentrated extract was obtained. The extract was then weighed and stored in amber-colored bottles at 4 °C for further analysis. The extraction efficiency was calculated using **Eq. (1)** (Mokaizh et al., 2025).

(1) $$\text{Extract yield}\;\left(\%\right)=\left(\frac{\mathrm{Dry}\;\mathrm{weightof extracted product}}{\mathrm{weight}\;f\;\mathrm{plant material}}\right)\times 100$$

### 2.5 Phytochemical analysis

#### 2.5.1 TPC analysis

The TPC analysis of the extract was determined using a modified version of the Folin-Ciocalteu (FC) method. In this assay, 200 μL of the plant extract was mixed with 1 mL of 10 % FC reagent and allowed to react for 4 min. Subsequently, 800 μL of 7.5 % sodium carbonate solution was added to neutralize the mixture. The reaction mixture was then incubated at ambient temperature for 30 min. Absorbance was recorded at 760 nm using a UV–Visible spectrophotometer. Gallic acid served as the reference standard, and the results were expressed as milligrams of gallic acid equivalents per gram of extract (mg GAE/g), following the method of Joshi et al. (2023).

#### 2.5.2 DPPH free radical scavenging activity

Antioxidant capacity of the extract was tested using the DPPH radical scavenging assay. A stock solution was prepared by dissolving 2 mg of DPPH reagent in 5 mL of ethanol. 1 mL of this DPPH solution was then mixed with 3 mL of the plant extract at varied concentrations. The combination was incubated in darkness at 25 °C for 30 min before being measured at 517 nm with a spectrophotometer (Baliyan et al., 2022). Antioxidant activity was represented as the % of radical scavenging, determined using the following **Eq. (2)**:

(2) $$\text{Free radical scavenging}\%=\frac{\mathrm{OD}\;\text{of Control}-\mathrm{OD}\;\text{of Sample}}{\mathrm{OD}\;\text{of Control}}\times 100$$

#### 2.5.3 TFC analysis

The TFC was determined using the aluminium chloride colorimetric method. In this procedure, 1 mL of the plant extract was mixed with 3 mL of 99 % ethanol, 0.2 mL of 1 M potassium acetate, and 5 mL of 10 % aluminium chloride solution. To complete the reaction mixture, 6 mL of distilled water was added. The mixture was then allowed to incubate at room temperature for 30 min. Absorbance was recorded at 415 nm using a spectrophotometer. Quercetin was used as the standard, and the flavonoid content was expressed as milligrams of quercetin equivalent per gram of extract (mg QUE/g), as described by de Lima et al. (2024).

#### 2.5.4 Total chlorophyll content

A 1 mL aliquot of the plant extract (10 mg/mL) was mixed with 9.9 mL of 80 % acetone, and the resulting solution was centrifuged at 2000 rpm for 15 min (Akdas & Bakkalbasi, 2017). The absorbance was recorded at 645 nm and 663 nm, and the total chlorophyll content was quantified using **Eq. (3):**

(3) $$\mathrm{Total Chlorophyll}=\left(20.2\times \mathrm{Absorbance}\;\mathrm{at}\;645\right)+\left(8.02\times \mathrm{Absorbance}\;\mathrm{at}\;663\right)$$

### 2.6 Color analysis

The color of *Piper betel* L. extract powder was analyzed using a Lovibond Tintometer based on the CIELAB color space (L*, a*, b*). Before measurement, the instrument was calibrated using a standard white tile under controlled D65 lighting conditions. Approximately 2–3 g of the dried extract powder was weighed and evenly spread in an opaque sample cup to create a uniform surface, avoiding any irregularities (Zhbanova, 2020). The CIELAB system quantifies color using three coordinates:

• L*: Lightness (0 = black, 100 = white)
• a*: Green–Red axis (− = green, + = red)
• b*: Blue–Yellow axis (− = blue, + = yellow)

### 2.7 Analytical characterization of extract

#### 2.7.1 Fourier transform infrared spectroscopy (FTIR) analysis

The functional groups seen in the ***Piper betel*** L. extract were determined using FTIR (Parkin Elmer Spectrum −2 FTIR spectrometer) over a spectrum range of 4000 to 450 cm^−**1**^. Specific functional groups were identified based on FTIR peak data (Nasution & Wulandari, 2021).

#### 2.7.2 GC–MS analysis

The bioactive compounds in the extract were identified using GC–MS analysis (PerkinElmer GC–MS Clarus 680 system). Helium was the carrier gas, with a flow rate of 1.1 mL/min. The column temperature may be set anywhere between 70 °C and 280 °C. To validate the discovered compounds, the respective peak areas and retention durations were compared to the NIST and Wiley databases (McGlynn et al., 2025).

#### 2.7.3 SEM analysis

The surface characterization of the extract powder (150 μm particle size) was investigated using SEM (JSM 6490 LV, JEOL, Japan). The sample was mounted on double-sided carbon tape, which was attached to metal stubs. These stubs are coated with gold palladium sputter-coater (Model JFC-1600, JEOL, Japan at 20 mA) and viewed in SEM Bisht, Verma, et al. (2023).

### 2.8 Preparation of bacterial culture

The antibacterial activity of *Piper betel* L. leaf extract was assessed against *Bacillus cereus*, *Bacillus pumilus*, *Klebsiella pneumoniae*, and *Escherichia coli* under optimized extraction conditions. The bacterial strains were procured from the Department of Biosciences, Integral University, Lucknow, with accession numbers MTCC-160 (*B. pumilus*), MTCC-1305 (*B. cereus*), MTCC-109 (*K. pneumoniae*), and ATCC-25923 (*E. coli*). Stock cultures were initially maintained on nutrient agar (Merck) and activated in nutrient broth (Merck) by incubating at 37 °C for 24 h, then stored at 4 °C for future use. For antibacterial assays, each strain was inoculated into 100 mL of nutrient broth and incubated at 37 °C with shaking (200 rpm) for 20 h to achieve optimal growth. The cultures were centrifuged at 5000 rpm for 10 min, and the supernatant was discarded. The bacterial pellet was washed thrice with sterile saline solution to remove residual medium. The final suspension was adjusted to an optical density (OD) of 0.5–1 at 500 nm, corresponding to approximately 5 × 10^7^ CFU/mL, as described by Nayaka et al. (2021). To ensure experimental consistency, the bacterial suspensions used for antimicrobial assays were further standardized to a concentration of 1 × 10^6^ CFU/mL by adjusting the turbidity to match a 0.5 McFarland standard before inoculation. This standardization ensured uniform bacterial load across all treatments. The actual CFU/mL was validated by serial dilution and plating 100 μL onto nutrient agar plates, followed by incubation at 37 °C for 24 h. Colonies were manually counted, and CFU/mL was calculated using **Eq. (4)**:

(4) $$\frac{\mathrm{CFU}}{\mathrm{mL}}=\frac{\mathrm{No}\;\mathrm{of colonies}\times \mathrm{Dilution factor}}{\mathrm{Volume plated}\;\left(\mathrm{mL}\right)}$$

#### 2.8.1 Determination of antibacterial activity

The antibacterial properties of *Piper betel* L. extract were evaluated using the agar well diffusion method. Sterilized nutrient agar (NA) was poured into sterile petriplates and allowed to solidify under aseptic conditions. A standardized bacterial suspension (1 × 10^8^ CFU/mL) was evenly spread on the surface of each plate using a sterile spreader. Wells of 6 mm diameter were created in the agar using a sterile cork borer. Each well was loaded with 50 μL of *Piper betel* ethanol extract. Plates were incubated at 37 °C for 24 h. A 5 % DMSO solution served as the negative control, while ampicillin was used as the positive control. All experiments were conducted in triplicate to ensure reproducibility. The antibacterial activity was assessed by measuring the diameter of the inhibition zones (in mm) formed around the wells after incubation, as described by Jamelarin and Balinado (2019).

### 2.9 Statistical analysis

Design-Expert Software (Version 13.0.5) for Response Surface Methodology (RSM) performed all analyses of all experimental data. Extensive statistical relevance of extraction parameters was found by analysis of variance (ANOVA) at 1 % and 5 % significance levels for model validation (Singh et al., 2019).

## 3 Results and discussions

The extraction process of phytochemicals from *Piper betle* L. leaves with MAE was thoroughly investigated and optimized in this study. To optimize this process, extraction time, microwave power, and solid-to-solvent ratio at a constant particle size of 150 μm and a fixed sample size of 10 g were systematically evaluated for maximizing phytochemical yield. To achieve optimization, the study used the Box–Behnken Design (BBD) by using the RSM framework with 17 experimental runs. This approach was systematically used to design and conduct experiments, and the extracted data went through both statistical and graphical analysis to validate the model significance. The properties of *Piper betle* L. extract has been studied in two different stages: 1) Optimization was performed for the extraction conditions to maximize the yield of phytochemicals, especially volatile bioactive compounds from dried betel leaf powder. Moreover, the phytochemical composition of the obtained extract was qualitatively characterized, and the extract yield was obtained quantitatively, 2) In the second phase, this optimized extract was further used to characterize its physicochemical properties as well as functional properties. In order to validate the model's accuracy, experimental tests were conducted at the optimized conditions, and the results were compared with the predicted values generated by the RSM model.

### 3.1 Analytical evaluation of betel leaf extract

The experimental data found in Tables 1, show the influence of each independent variable on the measured responses. Statistical adjustments were made to the results to end up with the most favorable extraction results **(S Table 2)**. The model proved to increase experimental and predicted values to a strong correlation with the optimized extraction conditions, which demonstrate the efficacy and economy of the RSM model for the phytochemical extraction from *Piper betle* L.

**Table 1:** BBD-based optimization of MAE: Effect of Microwave Time, Power, and Solid-to-Solvent Ratio.

Run	Factors			Responses				
	Time (min)	Power (watt)	Solid: Solvent ratio (mg/mL)	Extract Yield (%)	TPC (mg/GAE/g)	TFC (mg/ QUE/g)	Antioxidant Activity (%)	Total Chlorophyll (mg/mL)
1.	1	240	10	11.2	89.79	40.96	64.56	27.4
2.	2	300	10	8.5	77.79	35.6	69.91*	33.6
3.	2	180	30	10.01	94.62	41.28	57.63	41.9
4.	2	240	20	10.89	88.52	48.03	58.89	55.69
5.	1	180	20	11.4*	98.39*	42.48	50.38**	34.9
6.	2	240	20	10.87	88.56	48	58.87	55.77
7.	3	300	20	8.67	74.56**	33.6**	54.57	26.2**
8.	1	300	20	9.7	79.82	34.6	69.1	36.5
9.	2	180	10	10.1	96.31	41.8	53.57	46.9
10	1	240	30	9.89	88.01	39.6	60.31	38.9
11.	2	240	20	10.79	88.49	48	58.92	55.78*
12	3	180	20	10.02	93.2	35	61.03	50.5
13.	2	240	20	10.82	88.6	48.1*	58.82	55.78*
14.	3	240	10	8.69	84.32	33.9	63.73	44.8
15.	2	300	30	8.19**	76.21	38.6	54.1	30.6
16.	2	240	20	10.81	86.34	48	58.85	55.12
17.	2	240	10	8.70	85.30	47	59.80	45.70

*: highest; **: lowest.

### 3.2 Extract yield

Maintaining a constant sample and particle size, the extraction yield of *Piper betle* L. leaf extract ranged between 8.19 % and 11.4 %, with the highest yield (11.4 %) obtained under the conditions of microwave power (180 W), extraction time (1 min), and a solid-to-solvent (1:20). On all other parameters kept constant, the lowest yield (8.19 %) was found under the conditions of 300 W microwave power, 2 min extraction time, and a solid-to-solvent ratio of 1:30. These findings imply that maximizing extraction efficiency depends much on optimizing microwave power and solvent ratio.

Achieving greater yields (11.4 %) within much less processing time, MAE showed better efficiency than conventional soxhlet extraction (Muruganandam et al., 2017), hence minimizing energy usage and solvent waste. Significant impacts of both linear and quadratic elements on extraction yield were found by statistical analysis, applying a regression model. Moreover, significant interactions (*p* < 0.05) were found between solid-to-solvent ratio and power, microwave power and time, and solid-to-solvent ratio and time **(S Table 2)**.

**Fig. 1:**
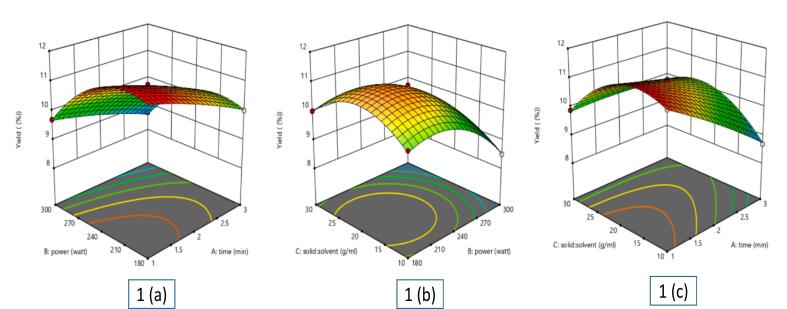
Effect of various independent factors on betel leaf extract yield: (a) Influence of power and time; (b) Influence of solid-liquid ratio and power; (c) Influence of solid: solvent ratio and time.

Three-dimensional (3D) surface plots were created to show how independent variables affect extract yield and provide insightful analysis of response variances **(**Fig. 1a, b, and c**)**. The 3D plots showed that extract yield first rose with increasing microwave power, but yield fell off above 240 W. This decline could be ascribed to warming, which can cause bioactive chemicals to degrade or undergo thermal breakdown. For extraction time, a corresponding trend was noted. Increased solvent penetration and mass transfer first raised the yield, but extended microwave radiation reduced the yield. As extraction equilibrium was attained, either solvent evaporation or less interaction between solute and solvent could be the cause of this drop. Furthermore, clearly affecting extraction efficiency was the solid-to-solvent ratio. Solvent availability was limited at smaller ratios, therefore limiting solute diffusion. But with too high solvent volumes, microwave energy dispersion per unit mass dropped, hence lowering extraction efficiency. ANOVA **(S Table 2)** helped to validate the model. The substantial *p*-value of 369.34 (*p* < 0.001) produced by the statistical study shows the great dependability of the model.

### 3.3 Impact on TPC analysis

With notable differences across several extraction settings, the TPC values ranged from 74.56 to 98.39 mg GAE/g **(**Table 1**)**. Whereas the lowest value (74.56 mg GAE/g) was obtained at 300 W microwave power, 3 min extraction time, and the same solid-to-solvent ratio of 1:20, the highest TPC value (98.39 mg GAE/g) was recorded under conditions of 180 W microwave power, 1 min extraction time, and a solid-to-solvent ratio of 1:20. These findings emphasise the important part extraction conditions play in optimizing phenolic component retention.

**Fig. 2:**
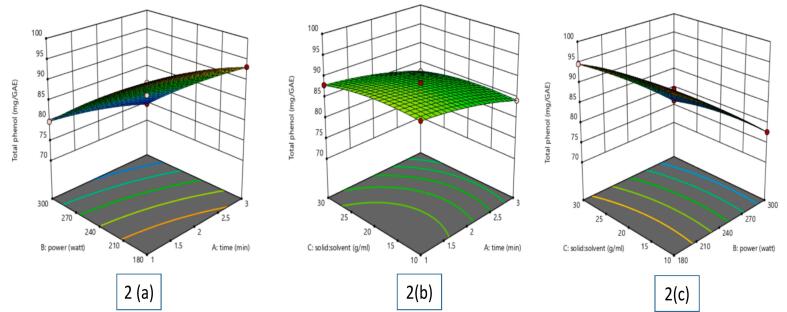
Effect of various independent factors on TPC extracted from betel leaf: (a) Influence of power and time; (b) Influence of solid-liquid ratio and time; (c) Influence of solid: solvent ratio and power.

In recovering phenolic compounds, MAE showed better efficiency than conventional extraction techniques like soxhlet and maceration, therefore generating noticeably higher TPC values (Das et al., 2019). Using 3D response surface plots **(**Fig. 2(a), 2(b), 2(c)**)**, the effects of extraction time, microwave power, and solid-to-solvent ratio on TPC were further investigated. The graphs showed a negative association between TPC and extraction time, meaning that increasing the length of time spent extracting gradually reduced phenolic content. For microwave power, a similar tendency was seen whereby raising the power level produced lower TPC values.

TPC was somewhat influenced by the solid-to-solvent ratio. With an increased solid-to-solvent ratio, a little increase in TPC was first seen; this resulted in a steady phase whereby additional modifications had little effect. But too high solvent volumes caused dilution effects for extended extraction durations, hence lowering the quantity of phenolic components in the extract.

Thermal degradation of phenolic compounds is the reason behind the drop in TPC at increasing microwave power levels. Strong microwave energy promotes molecular collisions and energy transmission, therefore upsetting the chemical bonds in the phenolic structures and causing their collapse. Furthermore, aggravating the breakdown of phenolic compounds is extended exposure to strong microwave power, which raises the possibility of oxidation and polymerisation events.

These results highlight the requirement of rigorous MAE parameter optimisation to reconcile effective extraction with compound stability. Although microwave energy improves the extraction of bioactive molecules, extended exposure and too high-power levels might cause major deterioration. Therefore, maximizing the preservation of important phenolic components in *Piper betle* L. extracts depends on using modest power settings and regulated extraction times.

### 3.4 Impact on TFC analysis

**Fig. 3:**
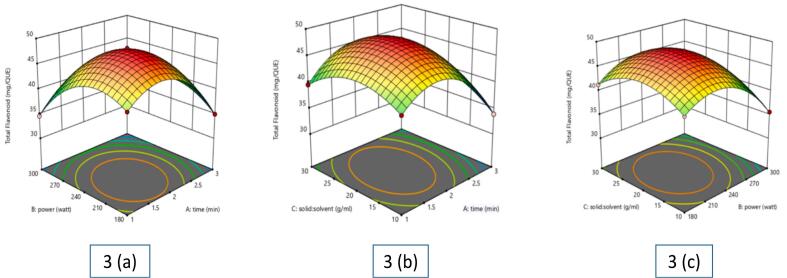
Effect of various independent factors on TFC extracted from betel leaf: (a) Influence of power and time; (b) Influence of solid-liquid ratio and time; (c) Influence of solid: solvent ratio and power

The results of TFC analysis of *Piper betle* L. extract ranged from 33.6 to 41.8 mg QUE/g **(**Table 1**)**. The lowest TFC (33.6 mg QUE/g) was recorded at 300 W, 3 min, 1:20 solid-to-solvent ratio, while the highest (48.1 mg QUE/g) was obtained at 240 W, 2 min, 1:20 solid-to-solvent ratio. MAE significantly improved flavonoid recovery as shown in Fig. 3 **(a, b, c)**. A triphasic extraction pattern was observed: (i) an initial rise due to microwave-induced cell wall breakdown, (ii) a plateau phase where extraction and degradation rates balanced, and (iii) a decline at higher microwave power (300 W) and longer times (3 min), likely due to thermal degradation or oxidation. The solid-to-solvent ratio initially enhanced extraction but later caused dilution. Moderate microwave power (240 W) optimized extraction, whereas excessive energy disrupted flavonoid stability (Morgan & Aboshanab, 2024). Ethanol and ethyl acetate proved effective solvents, with ethanol extracts confirming the presence of flavonoids, phenolics, and steroids. These findings highlight the importance of optimizing MAE parameters to maximize flavonoid yield while preventing degradation, ensuring *Piper betle* L. extracts retain their bioactive potential for functional foods, nutraceuticals, and medicinal applications (Sharma & Janmeda, 2017).

### 3.5 Antioxidant activity

**Fig. 4:**
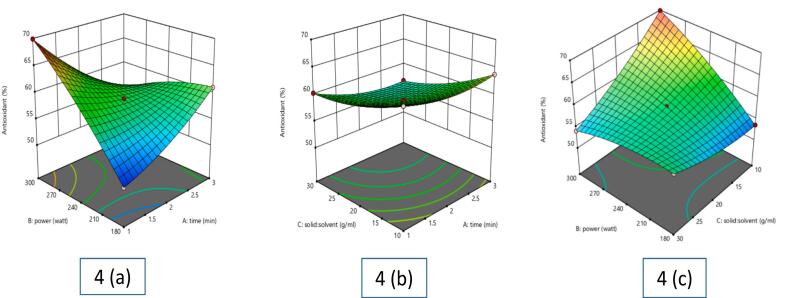
Effect of various independent factors on antioxidant activity of betel leaf extract: (a) Influence of power and time; (b) Influence of solid-liquid ratio and time; (c) Influence of solid: solvent ratio and power

The antioxidant potential of *Piper betle* L. extract was evaluated using the DPPH radical scavenging assay, which measures the extract's ability to neutralize free radicals. Results ranged from 50.38 % to 69.91 %, depending on extraction conditions **(**Table 1**)**. The highest activity (69.91 %) was observed at 300 W, 2 min, 1:10 solid-to-solvent ratio, while the lowest (50.38 %) occurred at 180 W, 1 min, 1:20 ratio. MAE enhanced antioxidant yield as shown in Fig. 4 **(a, b, c)**. The DPPH assay confirmed that higher microwave power initially improved extraction by breaking cell walls and releasing bioactive compounds. However, prolonged exposure led to degradation of heat-sensitive antioxidants, reducing radical scavenging activity. An inverse relationship was noted between the solid-to-solvent ratio and antioxidant activity, indicating dilution effects at higher ratios. Statistical analysis (*p* < 0.05) validated these trends, showing a significant correlation between antioxidant activity and extraction parameters. These findings highlight the importance of optimizing MAE conditions to maximize antioxidant yield while preventing degradation, ensuring *Piper betle* L. extract retains its bioactive potential for functional foods, nutraceuticals, and pharmaceuticals.

### 3.6 Total chlorophyll content

**Fig. 5:**
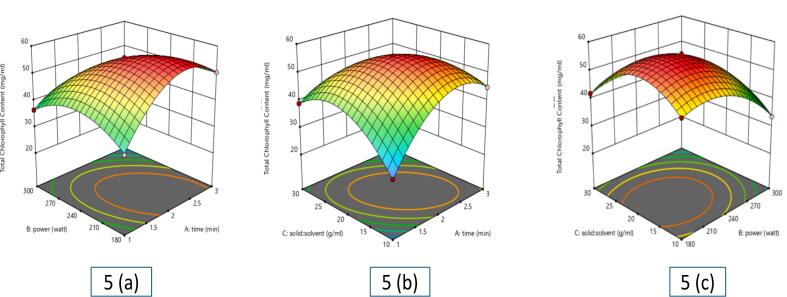
Effect of various independent factors on chlorophyll content of betel leaf extract: (a) Influence of power and time; (b) Influence of solid-liquid ratio and time; (c) Influence of solid: solvent ratio and power.

The chlorophyll concentration in *Piper betel* L. extracts was determined using spectrophotometric analysis, with variations observed based on MAE conditions as illustrated in Fig. 5**a, b, c**. The highest chlorophyll content (55.78 mg/mL) was achieved under 240 W microwave power, a 2 min extraction time, and a 1:20 solid-to-solvent ratio, while the lowest (26.2 mg/mL) was recorded at 300 W, 3 min, and 1:20 solid-solvent ratio. Extraction time initially increased chlorophyll yield, indicating effective pigment release, but prolonged exposure led to heat-induced degradation. Similarly, microwave power beyond 240 W accelerated chlorophyll breakdown, particularly affecting chlorophyll *a*, due to thermal stress. The solid-to-solvent ratio exhibited a saturation effect, where an optimal solvent volume enhanced solubility, but excessive solvent caused dilution, reducing extraction efficiency. Statistical analysis (*p* < 0.05) confirmed a strong correlation between extraction parameters and chlorophyll yield, with a reliable predictive model.

### 3.7 Optimization and validations

Design Expert Software optimized the extraction parameters for *Piper betle* L. bioactive components using RSM and BBD. The optimal conditions, 239.6 W microwave power, 1.588 min extraction time, and a 1:22 solid-to-solvent ratio were validated through experimental trials, closely matching predicted values **(S Table 3)**. Chlorophyll content (42.021 mg/mL) was nearly identical to the expected 42.936 mg/mL, while extract yield (8.92 %) closely aligned with the predicted 9.403 %. Similarly, total phenolic content (77.982 mg GAE/g), flavonoid content (38.991 mg QUE/g), and antioxidant activity (62.95 %) showed minimal deviations from their projected values. Statistical validation confirmed the model's accuracy (*p* < 0.05), with an insignificant lack-of-fit test, ensuring high predictive reliability. The study highlighted the role of controlled microwave power in enhancing solvent penetration and cell wall disruption, while excessive energy input risked thermal degradation of bioactive molecules.

### 3.8 FTIR analysis

**Fig. 6:**
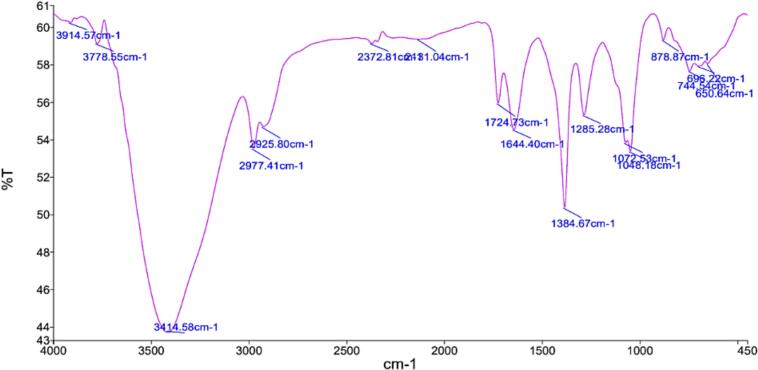
FTIR spectrum of betel leaf extract

The *Piper betel* L. extract'sS FTIR spectrum exposed several functional groups, therefore verifying the complex phytochemical composition of the material (Fig. 6, S Table 4). Characteristic of phenolic and hydroxyl compounds, the spectrum analysis revealed a clear alcohol (O—H) stretching vibration at 3678.55 cm^−1^ together with a broad peak at 3414.58 cm^−1^, showing hydrogen-bonded O—H stretching. Furthermore, supporting the existence of alkane and alkene groups were the observations of sp^2^ and sp^1^ hybridised C—H stretching vibrations at 2977.41 cm^−1^ and 2925.80 cm^−1^ respectively. These results match previously recorded FTIR analysis of *Piper betle* L. extracts (Elkobrosy et al., 2023). Moreover, a strong carbonyl (C**=**O) absorption peak at 1724.73 cm^−1^ indicated ketones, aldehydes, and carboxylic acids; a prominent absorption at 1644.40 cm^−1^ was ascribed to alkene (C**=**C) stretching vibrations, generally found in flavonoids and other polyphenolic compounds. Further confirming the existence of bioactive phenolic compounds was a medium-intensity band at 1384.64 cm^1^ corresponding with O—H bending of phenolic groups. Confirming the presence of amine (-NH) groups, ether/ester (C—O) linkages, and phenol alcohol (—OH) functional groups all of which help to explain *Piper betel's* antioxidant and medicinal qualities other peaks were noted. These several functional groups fit the well-documented pharmacological characteristics of the plant, therefore supporting the possible uses of *P. betle* extract in cosmetic, nutraceutical, and pharmaceutical formulations.

### 3.9 SEM analysis

**Fig. 7:**
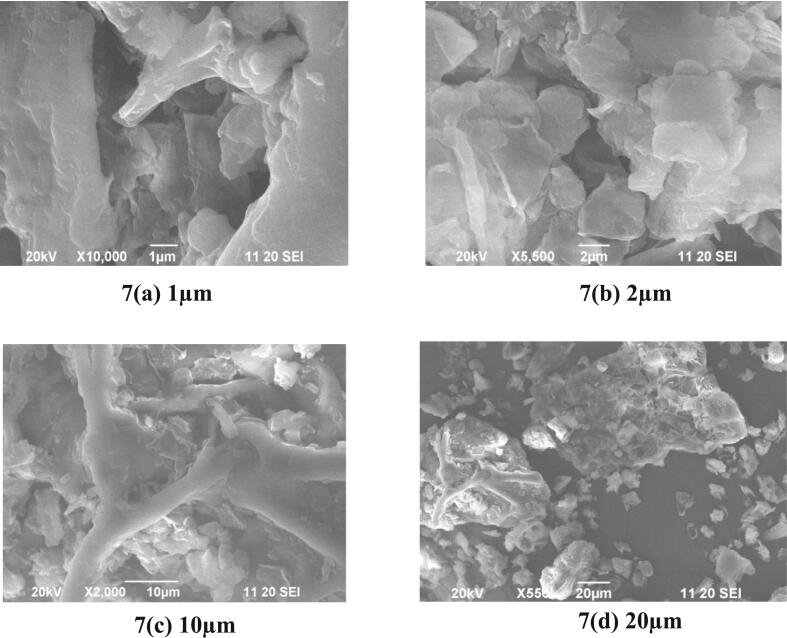
SEM analysis of betel leaf under optimal microwave extraction conditions

SEM analysis revealed significant morphogical alterations in *Piper betel* L. leaves subjected to MAE, underscoring the technique's efficiency **(**Fig. 7**)**. The micrographs demonstrated increased porosity, surface roughness, cellular deformation, fissures, and exposed vascular bundles, showing enhanced solvent penetration and improved phytochemical release. At ×2000 magnification (10 μm resolution, Fig. 7c), the SEM image showed expanded pores and disrupted cell walls, suggesting efficient breakdown of structural barriers under optimized MAE conditions. Unlike conventional soxhlet extraction, which relies on prolonged thermal exposure, MAE utilizes rapid dielectric heating and localized internal pressure to accelerate cellular rupture and solute diffusion, highlighting the ability to preserve bioactive compounds while maximizing extraction yield (Astyka et al., 2024).

### 3.10 GC–MS analysis

Based on their retention time (RT) and peak strength (S Fig. 1), the GC–MS study of ***Piper betle*** L. extract produced a comprehensive chemical profile including several bioactive chemicals. Multiple peaks on the GC–MS chromatogram, each belonging to a particular chemical, where peak intensity indicates the quantity of the compound and retention time aids in determining its chemical character. The table shows the found compounds together with their chemical formulae, structural features, and biological actions. The study turned up a wide spectrum of bioactive chemicals, including acids, alcohols, esters, long-chain hydrocarbons, alkaloids, amino, and nitro compounds, all of which add to the pharmacological possibilities of ***Piper betle*** L. extract. Neophytadiene had the greatest peak among the identified chemicals at RT = 30.309 min, suggesting its rather plentiful concentration in the extract. Physically active diterpene with anti-inflammatory, antioxidant, analgesic, antipyretic, and antibacterial actions is neophytadiene. Traditionally used also in treating rheumatism, skin conditions, and headaches, it is (Prasathkumar et al., 2022). With great antibacterial action against ***Staphylococcus aureus*** and antioxidant and antinociceptive properties, another important molecule, phytol, highlighted medicinal potential. At RT = 33.794 min, the study also verified the existence of hexadecenoic acid, a well-known saturated fatty acid having anti-inflammatory, antibacterial, and antifungal activities, detected with a peak area of 5.17 %. Reported to have high anti-inflammatory, antioxidant, and antibacterial properties, 4-Allyl-1,2-diacetoxybenzene was also found at RT = 26.175 min with a peak area of 19.42 % (Madhumita et al., 2020). Further validating the anticancer potential of ***Piper betle*** L. extracts was the identification of the 12a-Hydroxydalpanol molecule at RT = 22.552 min with a 3.93 % peak area, renowned for its antitumor and antioxidant capabilities. Found at RT = 23.372 min with the highest peak area of 43.88 %, another important chemical, 3-Allyl-6-methoxyphenyl acetate, highlights its strong antibacterial and antioxidant properties, therefore contributing significantly to the bioactivity of the extract.

Moreover, 3,7,11,15-Tetramethyl-2-hexadecen-1-ol, found at RT = 31.254 min with an area of 2.70 %, showed excellent antibacterial and antioxidant qualities, thereby contributing to the general therapeutic worth of the extract. These bioactive molecules taken together prove *Piper betle* L. medicinal worth in nutraceutical, pharmacological, and antibacterial uses. The results of the GC–MS investigation not only support the conventional use of betel leaves in herbal medicine but also show their possibilities for generating natural therapeutic agents with antioxidant, anti-inflammatory, antibacterial, and anticancer characteristics.

### 3.11 Chromaticity analysis of the optimized extract

By employing the CIELAB color space, the color properties of the green dye extracted from *Piper betle* L. under optimal MAE conditions were investigated. Three criteria L (luminosity), a (red-green axis), and b (blue-yellow axis) qualify colours in this color space (Hong et al., 2015). L stands for brightness: values range from 0 (black) to 100 (white). While the b axis measures blue-yellow balance, where positive values indicate yellow hues and negative values indicate blue hues, the a axis measures red-green balance. Here, positive values indicate red hues and negative values indicate green hues. As values stray from the neutral centre point, the color intensity rises. With “L*” value of 62.8*, the dye lies in the mid-range of brightness rather than being either quite dark or quite light. Negative values of −32.1* on “a*” coincide with green hues*, thereby confirming the natural green pigment of betel leaves. Positive values of 62.9* on the “b*“axis indicate yellow hues. The efficacy of the microwave-assisted extraction method, which effectively retained the natural green pigment while maintaining bioactive compounds, is validated by the chromaticity study.

### 3.12 Antibacterial activity

The antibacterial efficacy of *Piper betle* L. leaf extract was assessed against four pathogenic bacteria**,** with the inhibition zone measurements recorded at three different concentrations (50 mg/mL, 75 mg/mL, and 100 mg/mL) and compared with the standard antibiotic Ampicillin **(S Table 5)**.

The results demonstrate that the betel leaf extract showed notable antibacterial activity with the highest substantial inhibition zone recorded against *B. pumilus* (3.6 mm at 100 mg/mL), followed by *E. coli* (3.0 mm), *B. cereus* (2.9 mm), and *K. pneumoniae* (2.9 mm). Rich phenolic content of the extract explains its antibacterial action since it has been extensively recorded to have antimicrobial effects by disturbing bacterial cell membranes and enhancing permeability (Lobiuc et al., 2023). Compared with previous studies, where MAE using an ethanolic solvent yielded the most prominent inhibitory zones, betel leaf extract's antibacterial activity was further evaluated with traditional Soxhlet extraction methods. The highest inhibitory activity found for *B. pumilus* was 32 mm, for *B. cereus* was 26 mm, for *E. coli* was 28 mm, and for *K. pneumoniae* was 25 mm (Me et al., 2021). These findings highlight how effectively MAE extracts bioactive antimicrobial molecules; results either matched or surpassed those of traditional antibiotics such as Ampicillin. Moreover, the antibacterial qualities shown in this work fit the mechanism of action of phytochemicals, which target metabolic pathways and bacterial cell walls, hence generating cell death (Elisha et al., 2022). Targeting Gram-positive (*B. cereus and B. pumilus*) as well as Gram-negative *K. pneumoniae, E. coli* helps to further establish the broad-spectrum antibacterial action of betel leaf extract.

## 4 Conclusion

This study successfully optimized the MAE of *Piper betel* L. phytochemicals using Design Expert Software, identifying ideal conditions that significantly improved yield and quality over traditional methods. Key bioactive compounds, including flavonoids, antioxidants, and phenols, were confirmed, reinforcing betel leaf's therapeutic potential. Optimized conditions enhanced extraction efficiency, reduced solvent use, and minimized processing time, making MAE an eco-friendly and economical approach. The optimized extraction yielded 8.92 % extract, with TPC of 77.98 mg GAE/g, antioxidant activity of 62.95 %, TFC of 38.99 mg QUE/g, and chlorophyll content of 42.02 mg/mL. SEM analysis revealed significant structural changes, validating efficient cell disruption. FTIR confirmed the presence of diverse functional groups, while GC–MS identified bioactive compounds with antibacterial, antioxidant, and anti-inflammatory properties. Antimicrobial assays demonstrated strong inhibition against *B. pumilus, B. cereus, E. coli*, and *K. pneumoniae*. The study highlights MAE as a sustainable and potent method for bioactive extraction, with promising applications in pharmaceuticals, nutraceuticals, and cosmetics. Further research could lead to new natural product-based health solutions. Further studies are recommended to evaluate the in vivo biological activities and toxicological profiles of the extracted compounds to ensure safety and efficacy. Scaling up the MAE process for industrial applications should be explored, along with encapsulation or formulation studies to enhance bioavailability and stability. Moreover, combining MAE with environmentally friendly solvents, such as green or deep eutectic solvents, has the potential to further enhance the ecological sustainability of the extraction process. Investigating the synergistic effects of betel phytochemicals with other plant-based bioactives could open new avenues in the development of functional foods, natural preservatives, and therapeutic agents.

## CRediT authorship contribution statement

**Tripti Singh:** Writing – original draft, Methodology, Investigation, Conceptualization. **Zaryab Shafi:** Writing – review & editing, Formal analysis. **Rahul Singh:** Supervision. **Bhawna Bisht:** Writing – review & editing. **Krishna Kumar Yadav:** Writing – review & editing. **Jari S. Algethami:** Writing – review & editing. **Ghadah Shukri Albakri:** Writing – review & editing. **Maha Awjan Alreshidi:** Writing – review & editing.

## Declaration of competing interest

The authors declare that they have no known competing financial interests or personal relationships that could have appeared to influence the work reported in this paper.

## Data Availability

Data will be made available on request.

## Appendix A Supplementary data

Supplementary material Image 1
