# Supplementary material for: Microwave-assisted extraction of phytochemicals from Piper betle L.: Optimization, characterization, and bioactivity evaluation
Source: Food Chem X. 2025 Jun 17;29:102672. doi: 10.1016/j.fochx.2025.102672 (PMC12221380; doi:10.1016/j.fochx.2025.102672)
Supplement: Supplementary file 1 — Supplementary material [file mmc1.docx]

**Appendix A Supplementary data**

**Microwave-Assisted Extraction of Phytochemicals from Piper betle L.: Optimization, Characterization, and Bioactivity Evaluation**

**Tripti Singh^a^, Zaryab Shafi^a^, Rahul Singh^b,*^, Bhawna Bisht^c,*^, Krishna Kumar Yadav^d,e^, Jari S. Algethami^f,g^, Ghadah Shukri Albakri^h^, Maha Awjan Alreshidi^i^**

^a^Department of Biosciences, Integral University, Lucknow, Uttar Pradesh-226026, India

^b^Department of Bioengineering, Integral University, Lucknow, Uttar Pradesh-226026, India

^c^Department of Food Science & Technology, Graphic Era (Deemed to be University), Dehradun-248002, India

^d^Department of VLSI Microelectronics, Saveetha School of Engineering, Saveetha Institute of Medical and Technical Sciences (SIMATS), Saveetha University, Chennai-602105, Tamil Nadu, India

^e^Environmental and Atmospheric Sciences Research Group, Scientific Research Center, Al-Ayen University, Nasiriyah, Thi-Qar-64001, Iraq

^f^Department of Chemistry, College of Science and Arts, Najran University, P.O. Box, 1988, Najran-11001, Saudi Arabia

^g^Advanced Materials and Nano-Research Centre (AMNRC), Najran University, Najran 11001, Saudi Arabia

^h^Department of Teaching and Learning, College of Education and Human development, Princess Nourah bint Abdulrahman University, P.O. Box 84428, Riyadh 11671, Saudi Arabia

^i^Department of Chemistry, College of Science, University of Ha’il, Ha’il 81441, Saudi Arabia

***Corresponding author Email Id’s:**

**Bhawna Bisht:** *bhawnabisht494@gmail.com*

**Dr. Rahul Singh:** *r*[*ahulsingh.jnu@gmail.com*](mailto:rahulsingh.jnu@gmail.com)

# **S Table 1. Parameters selected for preliminary trials for maximizing extract yield of *Piper betel* L.**

| **S. No.** | **Parameter** | **Level** | **Value of Level** |
| --- | --- | --- | --- |
| 1. | Solvent | 5 | Ethanol, Methanol, Hexane, Ethyl Acetate, Water |
| 2. | Particle size | 4 | 150 µm, 200 µm, 300 µm, 400 µm |
| 3. | Sample size | 4 | 2 g, 5 g,10 g,15 g |

**S Table 2. ANOVA-based assessment of the influence of process variables on extract yield, TPC, TFC, Total Chlorophyll, and Antioxidant Activity**

| **Parameters** | **Yield (%)** | | **TPC**  **(mg/GAE/g)** | | **TFC**  **(mg/GAE/g)** | | **Antioxidants**  **(%)** | | **Total Chlorophyll Content (mg/ml)** | |
| --- | --- | --- | --- | --- | --- | --- | --- | --- | --- | --- |
| **Source** | **F- value** | **p- value** | **F- value** | **p- value** | **F- value** | **p- value** | **F- value** | **p- value** | **F- value** | **p- value** |
| **Model** | 369.35 | <0.0001 | 150.74 | <0.0001 | 9106.72 | <0.0001 | 10215.33 | <0.0001 | 1114.41 | <0.0001 |
| 1. **Power** | 1085.44 | <0.0001 | 1224.18 | <0.0001 | 7064.82 | <0.0001 | 17148.58 | <0.0001 | 2018.11 | <0.0001 |
| 1. **Time** | 650.84 | <0.0001 | 101.42 | <0.0001 | 6374.52 | <0.0001 | 2181.24 | <0.0001 | 119.07 | <0.0001 |
| 1. **Solvent volume** | 9.33 | 0.0184 | 10.24 | 0.0151 | 559.39 | <0.0001 | 13697.80 | <0.0001 | 307.83 | <0.0001 |
| **AB** | 6.35 | 0.0398 | 0.6022 | 0.9640 | 1799.07 | <0.0001 | 16873 | <0.0001 | 1278.31 | <0.0001 |
| **AC** | 303.71 | <0.0001 | 0.0011 | 0.9743 | 1226.33 | <0.0001 | 424.73 | <0.0001 | 1167.31 | <0.0001 |
| **BC** | 2.51 | 0.1571 | 0.0054 | 0.9435 | 530.87 | <0.0001 | 10217.40 | <0.0001 | 4.49 | 0.0719 |
| **A^2^** | 8.18 | 0.0244 | 5.20 | 0.0567 | 2973.75 | <0.0001 | 1457.51 | <0.0001 | 2554.17 | <0.0001 |
| **B^2^** | 547.52 | <0.0001 | 4.53 | 0.0708 | 1941.71 | <0.0001 | 1236.74 | <0.0001 | 1966.46 | <0.0001 |
| **C^2^** | 622.54 | <0.0001 | 8.95 | 0.0202 | 8937 | <0.0001 | 1101.24 | <0.0001 | 2123.33 | <0.0001 |
| **R^2^** | 0.9979 | | 0.9949 | | 0.9999 | | 0.9995 | | 0.9989 | |
| **Adjusted R^2^** | 0.9952 | | 0.9883 | | 0.9899 | | 0.9988 | | 0.9875 | |
| **Predicted R^2^** | 0.9728 | | 0.9912 | | 0.9789 | | 0.9838 | | 0.9759 | |
| **Adequate precision** | 60.25 | | 41.54 | | 146.21 | | 99.34 | | 122.36 | |

**S Table 3. Comparison of predicted and experimental Data for extraction efficiency and bioactive compound yield in Piper betel L.**

| **Analysis** | **Predicted data** | **Experimental data** | **Relative Deviation (%)** |
| --- | --- | --- | --- |
| **Yield (%)** | 9.40 | 8.92 | 5.13 |
| **Total Chlorophyll (mg/mL)** | 42.93 | 42.02 | 2.13 |
| **TPC (mg/GAE)** | 78.97 | 77.98 | 1.26 |
| **TFC (mg/QUE)** | 39.84 | 38.99 | 2.13 |
| **Antioxidant Activity %** | 63.44 | 62.95 | 0.78 |

**S Table 4. Interpretation of FTIR spectrum absorptions, intensity, vibration, appearance, and bonds**

| **S. No** | **Absorption peak** | **Intensity** | **Appearance** | **Bond** | **Vibrations** | **Specific types of bonds** |
| --- | --- | --- | --- | --- | --- | --- |
| 1 | 3778.55 | Medium | Sharp | O-H | Stretching | Alcohol |
| 2 | 3414.58 | Medium | Broad | O-H | Stretching | Alcohol |
| 3 | 2977.41 | Strong | Sharp | C-H | Stretching | Alkane |
| 4 | 2372.81 | Strong | Sharp | O=C=O | Stretching | Carbon dioxide |
| 5 | 2181.04 | Weak | Straight | C≡C | Stretching | Alkyne |
| 6 | 1724.73 | Strong | Sharp | C=O | Stretching | Ketone |
| 7 | 1644.40 | Strong | Sharp | C=C | Stretching | Alkene |
| 8 | 1384.64 | Medium | Sharp | O-H | Bending | Phenol |
| 9 | 1285.28 | Strong | Sharp | C-O | Stretching | Aromatic ester |
| 10 | 1072.53 | Strong | Sharp | C-O | Stretching | Alkyl aryl ether |

####

#### **S Table 5. Evaluation of zone of inhibition (ZOI) for Piper betle L. extract against selected microbial strains**

| **S. No** | **Bacteria Name** | **Shape** | **Zone of Inhibition (ZOI)** | | | **ZOI due to Ampicillin** | **Time of Incubation** |
| --- | --- | --- | --- | --- | --- | --- | --- |
|  |  |  | **50 mg/mL** | **75 mg/mL** | **100mg/mL** |  |  |
| 1 | *B. pumilus* | Irregular | 1.9 | 2.7 | 3.2 | 3.6 | 48 h |
| 2 | *B. cereus* | Circular | 1.6 | 2.1 | 2.6 | 2.9 | 48 h |
| 3 | *K. pneumonia* | Irregular | 1.4 | 1.9 | 2.5 | 2.9 | 24 h |
| 4 | *E. Coli* | Circular | 1.8 | 2.1 | 2.8 | 3.0 | 24 h |

**
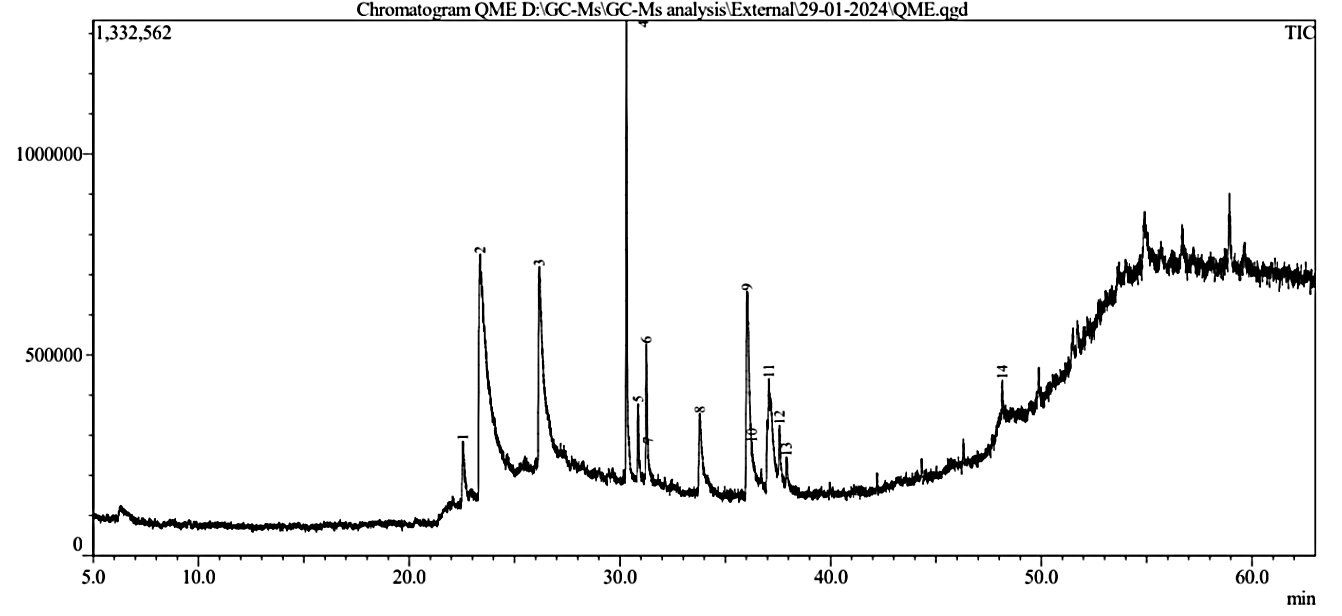
**

**S Fig 1. GC-MS analysis peaks of the optimized condition of MAE**
